# Wakefulness

**Published:** 1869-03

**Authors:** 


### WAKEFULNESS.

It is the complaint of many that they cannot go to sleep promptly on retiring; others are inclined to wake up during the night and find themselves unable to go to sleep again for some hours; a third class wake up early in the morning, feel as if they wanted and needed more sleep, but cannot get to sleep again, and finally get up unrefreshed, sad, or not in a very good humor. If sadly so, that this arises from a mind ill at ease, from sharp-pointed memories, from business entanglements, or the loss of the loved, we have no counsel to give just now; it was intended to make some suggestions which would avail to counteract the influence of physical causes or those of a constitutional character. In nearly all this class of cases, the immediate cause of the wakefulness is the attempt of the person to impose more sleep on the system than it requires under the circumstances. The body will no more take, naturally, a greater amount of sleep than is necessary for the wants of the system, than a gallon bottle will receive more fluid when its proper gallon has filled it to the brim. You may force sleep on nature by taking anodynes, but the sleep is not natural, and always fails to refresh. Each person requires an amount of sleep proportioned to his own constitution, temperament, occupation and age; the general rule is, that women require more sleep than men; all require more in winter than in summer; infancy requires more than mature life; and old age often less than either.

The great regulator of sleep is exercise; it is the best anodyne in the universe, and is the only one that is always safe, always efficient and always wholesome and natural. If you cannot take much exercise, take a little, and every second hour increase the distance, and soon you will be able to walk a mile more easily than you walked the first hundred yards. The following rules are of admirable utility: Arise and go to bed at a regular hour; avoid rigidly a moment's sleep in the day-time; get up as soon as you wake, even if it be in an hour; wash, dress and remain up until your regular hour of retiring, and you will seldom fail of two results within a week; you will go to sleep promptly, will sleep soundly, and will as certainly have as many hours sleep as you require under all the circumstances of your habits of life, temperament and muscular activities. Without these last, healthful, delightful, refreshing sleep is an impossibility.

